# Supplementary material for: Empirical prediction of variant-activated cryptic splice donors using population-based RNA-Seq data
Source: Nat Commun. 2022 Mar 29;13:1655. doi: 10.1038/s41467-022-29271-y (PMC8964760; doi:10.1038/s41467-022-29271-y)
Supplement: Supplementary file 1 — Supplementary Information [file 41467_2022_29271_MOESM1_ESM.pdf]

Supplementary Information

Empirical prediction of variant-activated cryptic splice donors using population-based RNA-Seq data

Ruebena Dawes[1,2] Himanshu Joshi[1], and Sandra T. Cooper[1,2,3*]

[1]Kids Neuroscience Centre, Kids Research, Children's Hospital at Westmead, Sydney, NSW2145, Australia

[2]Discipline of Child and Adolescent Health, Faculty of Health and Medicine, University of Sydney, Sydney, NSW2006, Australia

[3]The Children's Medical Research Institute, 214 Hawkesbury Road, Westmead NSW 2145, Sydney, Australia

This PDF File Includes:

Supplementary Figures 1-6

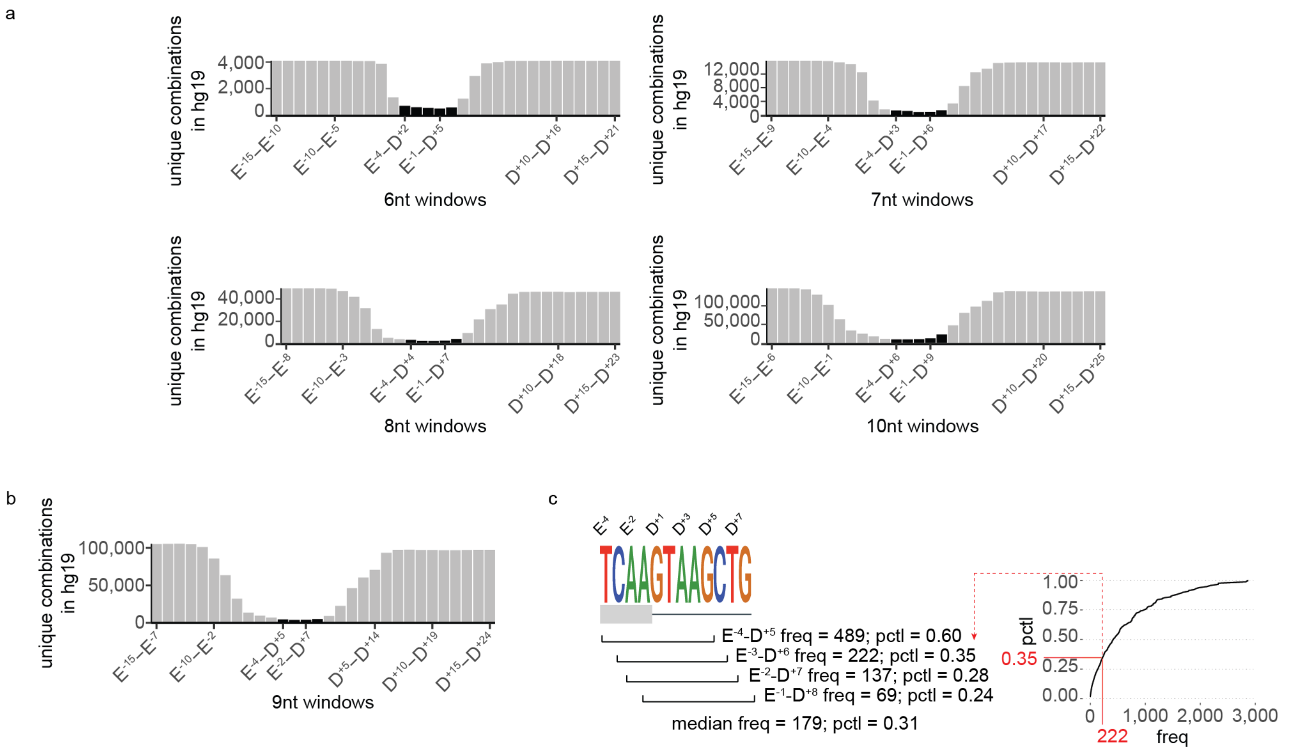

Supplementary Fig. 1 Calculation of Donor Frequency (DF) as a measure of donor strength. a-b) Frequency of unique combinations of donor sequences at each position of the exon-intron junction, spanning 6, 7, 8, 10 **(a)** or 9 **(b)** consecutive nucleotides. Black bars denote windows overlapping the E^{-4}-D^{+8} donor sequence window. Four sliding windows of 9 nt spanning the annotated-donor (coloured black), spanning 12 nt from the fourth-to-last exonic base (E^{-4}; E = exon) to the eighth intronic base (D^{+8}; D = donor), were used for DF calculation. These windows were chosen due to the jump in sequence diversity seen with windows upstream of/including E-5, and downstream of/including E+9. **c)** Donor Frequency is calculated as the median frequency across each 9nt window, converted to a cumulative percentile distribution. DF measures donor strength by how many annotated-donors in the human genome have the exact same sequence. In this example, a median DF raw value of 179 lies at the 31st percentile of a cumulative frequency distribution.

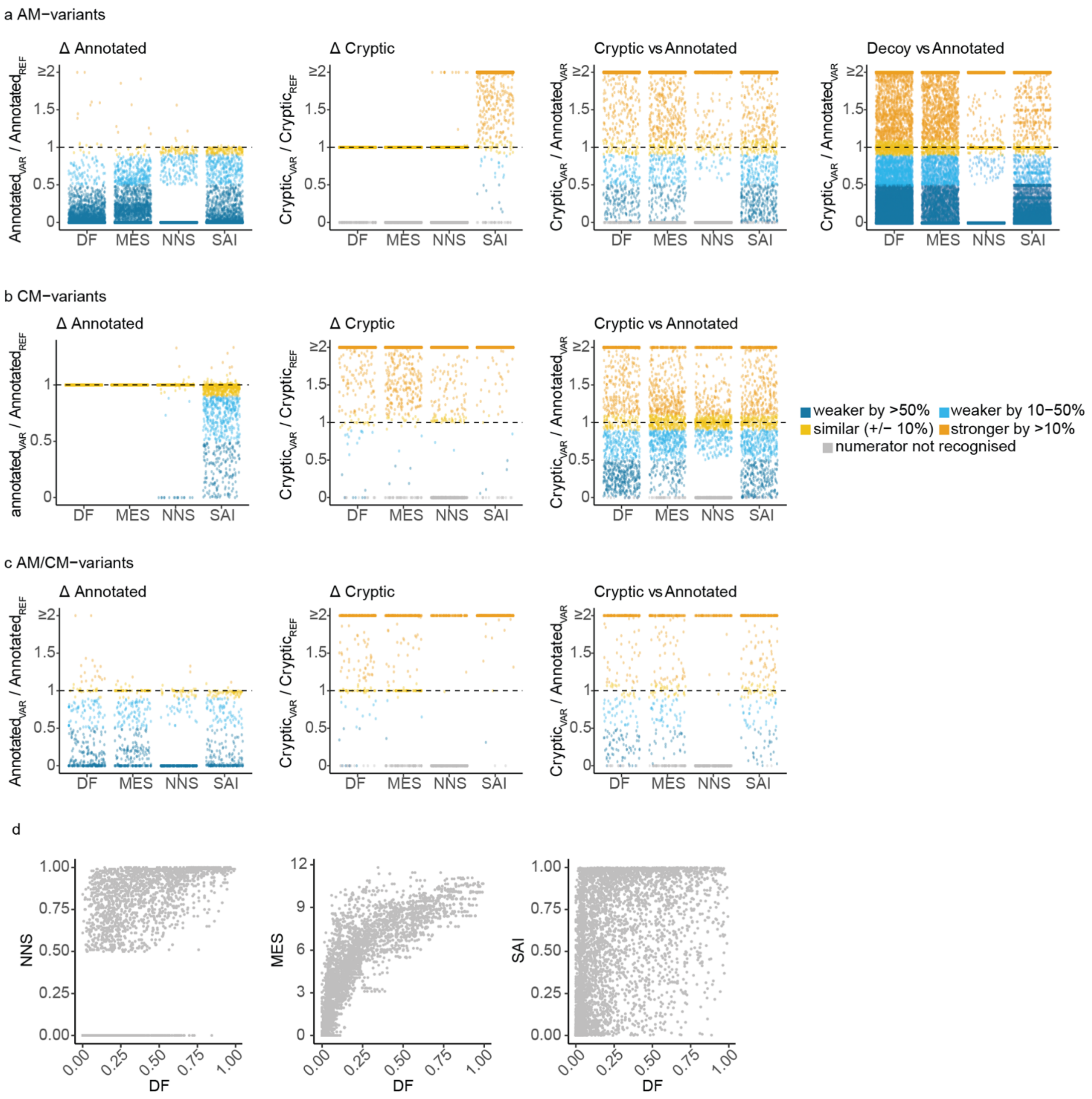

Supplementary Fig. 2 Algorithmic prediction of cryptic-activation. a-c) DF (Donor Frequency), MES (MaxEntScan), NNS (NNSplice) and SAI (SpliceAI) scores for **(a)** AM-variants **(b)** CM-variants and **(c)** AM/CM-variants. Colour coding is explained in the Figure key. When a donor strength score of 0 is returned, we set it to $1x10^{-6}$ to allow for the Δ calculations (VAR/REF; VAR = variant; REF = reference). **d)** Comparison of NNS, MES and SAI scores with DF for all cryptic-donors (scores for VAR sequence) in our Cryptic-Donor database. DF shows strongest correlation with MES. NNS does not recognise a subset of human donors to offer a strength prediction.

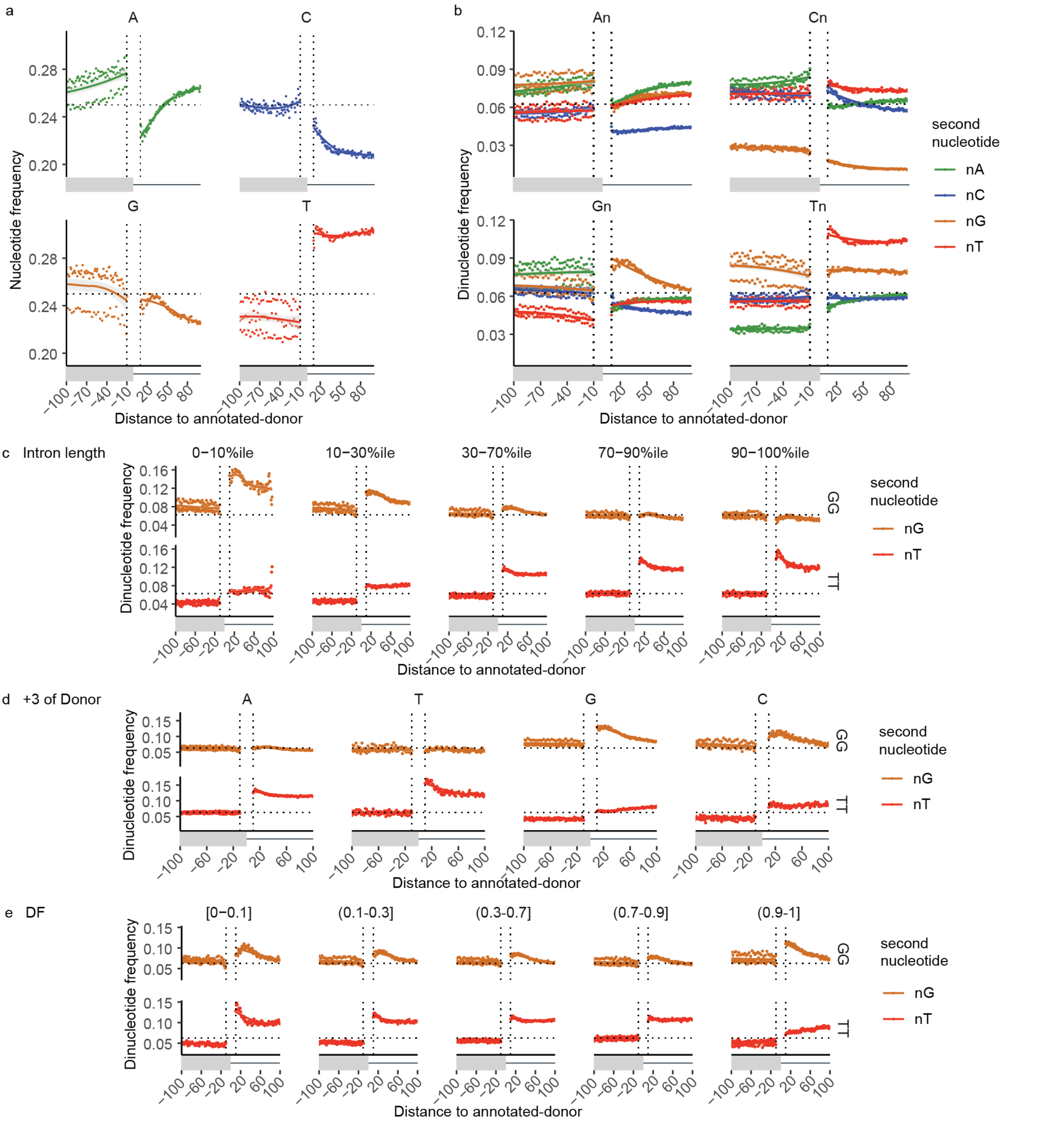

Supplementary Fig. 3 G- and T- dinucleotide repeats show distinct patterns of enrichment in different introns. a-b) frequencies of each **(a)** nucleotide and **(b)** dinucleotide at each position surrounding annotated-donors. Vertical dotted lines denote boundaries at -10 and +10 where calculations start (i.e. excluding the conserved extended splice-site region). Horizontal dotted lines denote a random frequency of **a)** 1/4 for single nucleotides and **b)** 1/16 for dinucleotides. Lines show LOESS smoothing (locally weighted smoothing i.e. trendlines) with confidence bands in grey. In **(b)** Panels are according to the first nucleotide and colours are according to the second nucleotide in the dinucleotide. Note enrichment of G- and T- dinucleotides in the first 50 nt of the intron. **c-e)** frequencies of dinucleotides GG, and TT at each position surrounding annotated-donors. Vertical dotted lines denote boundaries at -10 and +10 where calculations start, horizontal dotted line denotes a random frequency of 1/16. Lines show LOESS smoothing (locally weighted smoothing i.e. trendlines) with grey confidence bands. **a)** G-repeats are enriched in the shortest human introns whereas T-repeats are enriched in longer introns. Length bins: < 149 nt (< 10th percentile), 149-627 nt (10 - 30th percentile), 628-3010 nt (30 - 70th percentile), 3011-9270 nt (70 - 90th percentile), > 9270 nt (> 90th percentile). **b)** Annotated donors with D^{+3} G (or C) are enriched in G-dinucleotides whereas donors with D^{+3} A (or T) are enriched in T-dinucleotides. **c)** Rare donors (low Donor Frequency (DF)) show greater enrichment for T dinucleotide repeats compared with common donors (high DF).

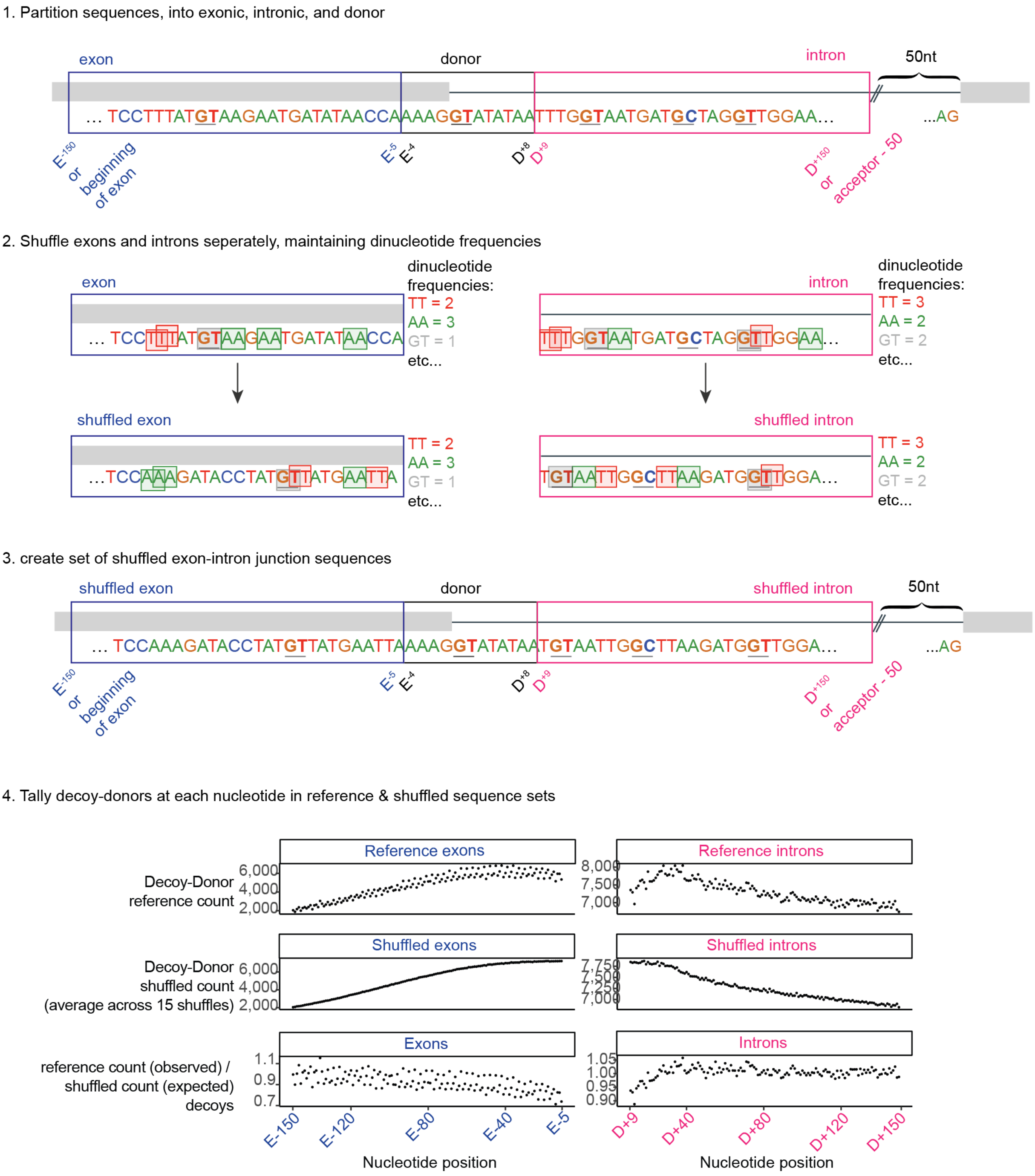

Supplementary Fig. 4 Schematic representation of how decoy depletion is calculated. Sequences in blue ‘exon’ and pink ‘intron’ boxes are shuffled separately (maintaining dinucleotide frequencies) and the number of actual decoy-donors at each position is divided by the number in the shuffled sequence set.

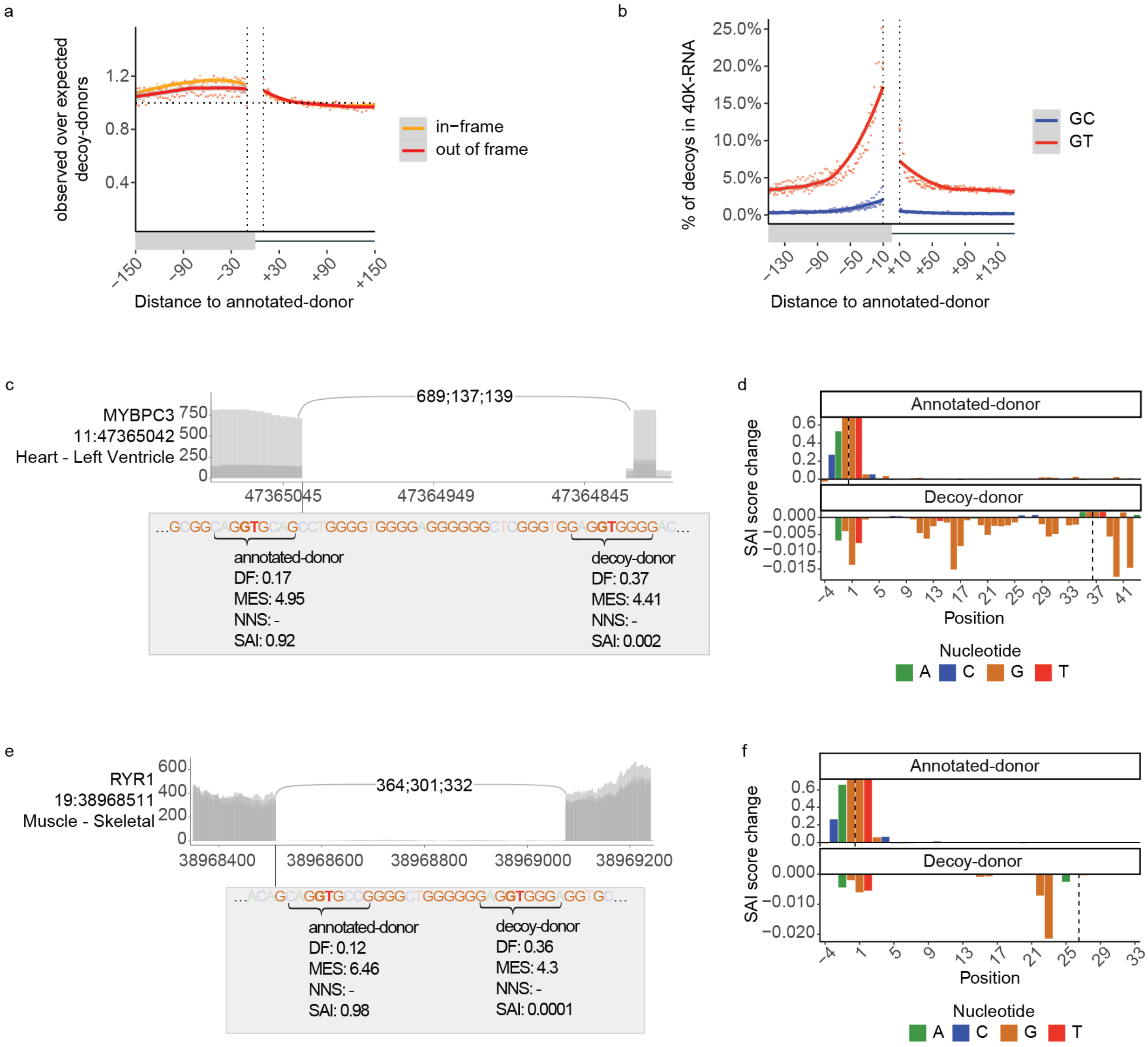

Supplementary Fig. 5 40K-RNA provides insight into natural use of decoy-donors by the spliceosome. a) The ratio of observed to expected 'GC' decoy-donors at each position +/-150 nt of annotated-donors. Yellow: In-frame exonic decoy-donors. Red: out-of-frame decoy-donors. **b)** The percent of decoys present in 40K-RNA +/-150 nt of annotated-donors. Red: 'GT' decoy-donors closer to the annotated-donor are more likely to be present in 40K-RNA. Blue: 'GC' decoy-donors are rarely seen in 40K-RNA. At each position the number of decoy-donors present in 40K-RNA is divided by the total number of naturally occurring decoy-donors at that position. Lines show LOESS smoothing (locally weighted smoothing i.e. trendlines) with grey confidence bands. **c-f)** two examples of decoy-donors overlapping G-repeats that outcompete the annotated-donor according to Donor Frequency (DF) and MaxEntScan (MES), but are not present in 40K-RNA, and which SpliceAI (SAI) correctly identifies as non-functional donors. NNS = NNSplice **c,e)** Shows overlays of 3 GTEx RNA-seq samples from the tissues with the highest TPM for that gene. The numbers (e.g., 689;137;139) denote the detected reads in each respective sample for that splice-junction. Algorithmic strength scores for annotated- and decoy-donors are boxed. **d,f)** Result of SAI in silico mutagenesis showing the bases contributing to predicted strength of the annotated-donor (top) and decoy-donor (bottom). 'SAI score change' denotes the decrease (if positive) or increase (if negative) on the predicted strength of the donor when that nucleotide is mutated (see methods). Black dashed line indicates the position of the annotated- or decoy-donor and position on the x-axis denotes the position of the nucleotide relative to the annotated-donor.

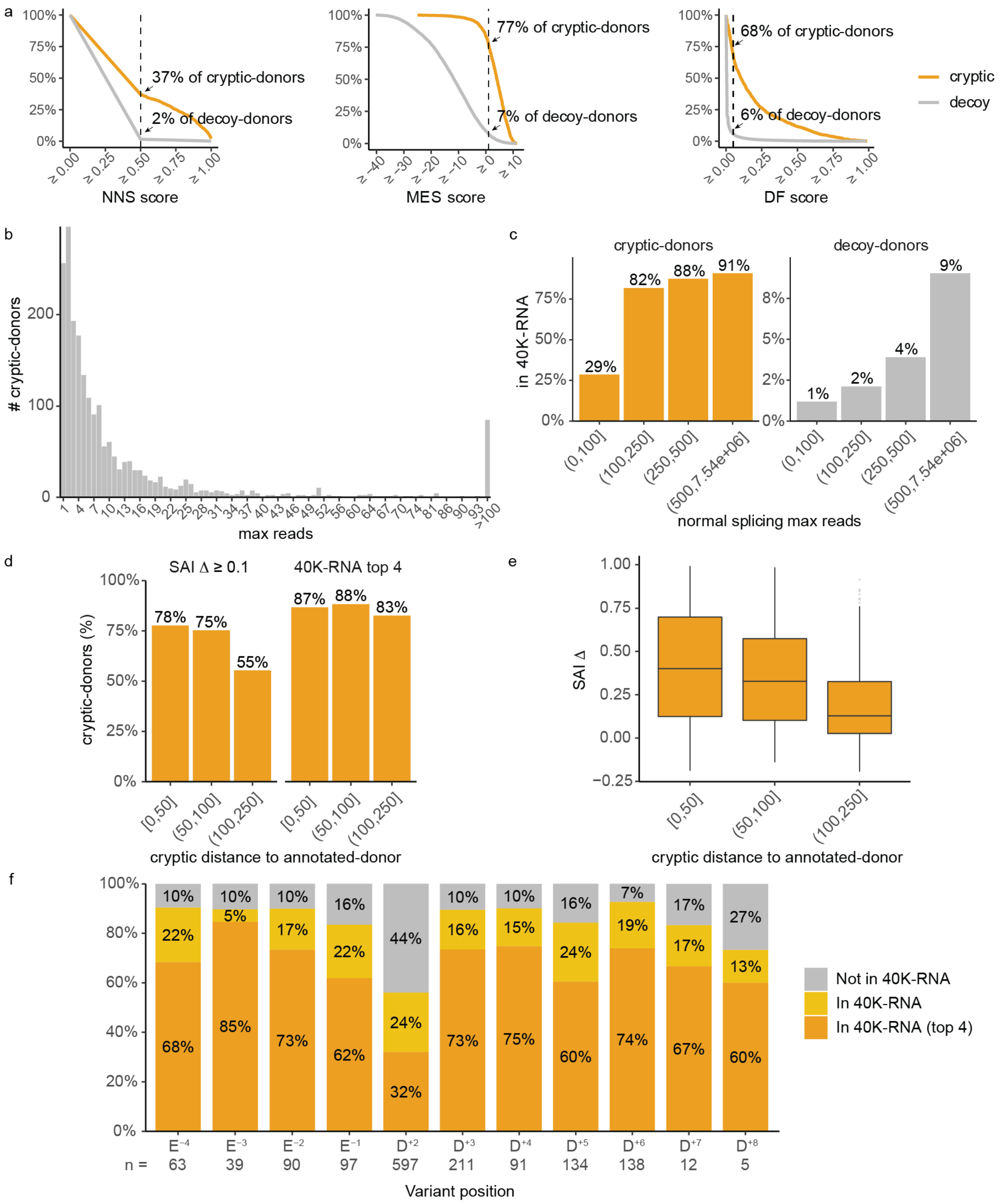

Supplementary Fig. 6 Effectiveness of 40K-RNA and SAI for prediction of cryptic-donor selection. a) Sensitivity (orange) and specificity (grey) of NNSplice (NNS) using a cut-off of 0.5, MaxEntScan (MES) using a cut-off of 1 and Donor Frequency (DF) using a cut-off of 0.05 to predict cryptic-donor activation in AM-variants. **b)** The maximum number of reads detected in any one RNA-seq sample across 40K-RNA for each cryptic-donor activated by an AM-variant. **c)** Only 29% of cryptic-donors for target genes with < 100 max reads corresponding to normal splicing are present in 40K-RNA, rising sharply to > 82% sensitivity for transcripts with more than 100 max reads corresponding to normal splicing. **d)** Percent of AM-variant cryptic-donors with SAI Δ scores greater than or equal to 0.1 (left) or in the 40K-RNA top 4 (right) in different bins according to cryptic distance to the annotated-donor. SpliceAI's sensitivity for AM-variants drops to 55% for cryptic-donors more than 100 nt from the annotated-donor **e)** SAI Δ scores for AM cryptic-donors (n = 2,348) relative to their distance from the annotated-donor. Internal lines denote the median value, and the lower and upper limits of the boxes represent 25th and 75th percentiles. The whiskers extend to the smallest and largest values no further than 1.5 x inter-quartile range (IQR). **f)** The percent of CM- and AM/CM-variant cryptics detected in 40K-RNA, according to the position of the SNV within the extended splice-site region of the activated cryptic-donor.
