# Supplementary material for: Empirical prediction of variant-activated cryptic splice donors using population-based RNA-Seq data
Source: Nat Commun. 2022 Mar 29;13:1655. doi: 10.1038/s41467-022-29271-y (PMC8964760; doi:10.1038/s41467-022-29271-y)
Supplement: Supplementary file 3 — Reporting Summary [file 41467_2022_29271_MOESM3_ESM.pdf]

nature portfolio

Corresponding author(s): Sandra T. Cooper

Last updated by author(s): 31.1.22

Reporting Summary

Nature Portfolio wishes to improve the reproducibility of the work that we publish. This form provides structure for consistency and transparency in reporting. For further information on Nature Portfolio policies, see our Editorial Policies and the Editorial Policy Checklist.

Statistics

For all statistical analyses, confirm that the following items are present in the figure legend, table legend, main text, or Methods section.

n/a	Confirmed	
☐	☒	The exact sample size (*n*) for each experimental group/condition, given as a discrete number and unit of measurement
☐	☒	A statement on whether measurements were taken from distinct samples or whether the same sample was measured repeatedly
☒	☐	The statistical test(s) used AND whether they are one- or two-sided<br>*Only common tests should be described solely by name; describe more complex techniques in the Methods section.*
☒	☐	A description of all covariates tested
☒	☐	A description of any assumptions or corrections, such as tests of normality and adjustment for multiple comparisons
☐	☒	A full description of the statistical parameters including central tendency (e.g. means) or other basic estimates (e.g. regression coefficient) AND variation (e.g. standard deviation) or associated estimates of uncertainty (e.g. confidence intervals)
☒	☐	For null hypothesis testing, the test statistic (e.g. *F*, *t*, *r*) with confidence intervals, effect sizes, degrees of freedom and *P* value noted<br>*Give P values as exact values whenever suitable.*
☒	☐	For Bayesian analysis, information on the choice of priors and Markov chain Monte Carlo settings
☒	☐	For hierarchical and complex designs, identification of the appropriate level for tests and full reporting of outcomes
☐	☒	Estimates of effect sizes (e.g. Cohen's *d*, Pearson's *r*), indicating how they were calculated

Our web collection on statistics for biologists contains articles on many of the points above.

Software and code

Policy information about availability of computer code

Data collection	The variants used in the cryptic-donor database are provided in the Source Data file. 40K-RNA is available as a web-resource at: https://kidsneuro.shinyapps.io/splicevault-40k/ . Additionally, the full dataset is available under restricted access to limit hosting costs. Access can be obtained by creating a google cloud billing account and downloading at this link using google cloud tools- https://storage.googleapis.com/misspl-db-data/misspl_events_40k_hg19.sql.gz. The GTEx v8 data used in this study were obtained from dbGaP accession number phs000424.v8.p2 [https://www.ncbi.nlm.nih.gov/projects/gap/cgi-bin/study.cgi?study_id=phs000424.v8.p2]. Intropolis data used in this study were obtained from the dedicated GitHub repository https://github.com/nellore/intropolis.<br><br>To collect data in this study custom code written in R (version 3.6) was used (available at https://github.com/kidsneuro-lab/40K-RNA). Datamash v3, Snakemake and Python3 were used for GTEx file processing. R packages tidyverse 1.3.1, data.table 1.14.2, R.utils 2.11.0, RSQLite 2.2.9, BSgenome.Hsapiens.1000genomes.hs37d5, 0.99.1, futile.logger 1.4.3, DBI 1.1.2, stringr 1.4 were used in our custom code.
Data analysis	All code required to replicate figures in the study are available in a GitHub repository: https://github.com/kidsneuro-lab/cryptic_donor_prediction. Additionally, code required to create 40K-RNA is available in a separate repository https://github.com/kidsneuro-lab/40K-RNA.<br><br>To analyse data in this study custom code written in R (version 3.6) was used (available at https://github.com/kidsneuro-lab/cryptic_donor_prediction). R packages data.table 1.14.2, tidyverse 1.3.1, BSgenome.Hsapiens.1000genomes.hs37d5 0.99.1, expss 0.11.1, stringi 1.7.6, Biostrings 2.62.0, universalmotif 1.12.2, Rfast 2.0.4, TRAMPR 1.0-9, ggpubr 0.4.0, gridExtra 2.3, patchwork 1.1.1, ggcorrplot 0.1.3, RColorBrewer 1.1-2, ggrastr 1.0.1 and eulerr 6.1.1 were used in our custom code.

For manuscripts utilizing custom algorithms or software that are central to the research but not yet described in published literature, software must be made available to editors and reviewers. We strongly encourage code deposition in a community repository (e.g. GitHub). See the Nature Portfolio guidelines for submitting code & software for further information.

Data

Policy information about availability of data

All manuscripts must include a data availability statement. This statement should provide the following information, where applicable:

- Accession codes, unique identifiers, or web links for publicly available datasets
- A description of any restrictions on data availability
- For clinical datasets or third party data, please ensure that the statement adheres to our policy

The variants used in the cryptic-donor database are provided in the Source Data file. 40K-RNA is available as a web-resource at: link. Additionally, the full dataset is available under restricted access to limit hosting costs. Access can be obtained by creating a google cloud billing account and downloading at this link using google cloud tools- https://storage.googleapis.com/misspl-db-data/misspl_events_40k_hg19.sql.gz. The GTEx v8 data used in this study were obtained from dbGaP accession number phs000424.v8.p2 [https://www.ncbi.nlm.nih.gov/projects/gap/cgi-bin/study.cgi?study_id=phs000424.v8.p2]. Intropolis data used in this study were obtained from the dedicated GitHub repository https://github.com/nellore/intropolis.

Field-specific reporting

Please select the one below that is the best fit for your research. If you are not sure, read the appropriate sections before making your selection.

☒ Life sciences ☐ Behavioural & social sciences ☐ Ecological, evolutionary & environmental sciences

For a reference copy of the document with all sections, see nature.com/documents/nr-reporting-summary-flat.pdf

Life sciences study design

All studies must disclose on these points even when the disclosure is negative.

Sample size	The cryptic-donor database was collated from available data sources of cryptic-donor variants. It is the largest analyzed set of cryptic-donors the authors are aware of.
Data exclusions	For the cryptic-donor database, The following inclusion criteria applied: 1) Variants had to occur within E-4-D+8 of the annotated or the cryptic-donor, otherwise they were excluded as outside the bounds of this analysis. 2) annotated cryptic-donors were within the same exon/intron as the variant (i.e. between the 5' end of the exon and 3' end of the intron surrounding the affected donor). 3) The annotated cryptic-donor VAR sequence had to have an essential GT/GC dinucleotide at D+1/D+2, to minimise misannotated variants being included.
Replication	Replication of the key finding that the cryptic-donor(s) activated by a genetic variant is/are present as (rare) splice-junctions in RNA-seq data is provided by:<br><br>1) Concordant outcomes across > 5,000 variants activating cryptic donor use compiled from multiple sources.<br><br>2) Similar efficacy of the method of ranking the top 4 events using two sources of public RNA-seq data (GTEx and Intropolis).<br><br>3) Detection of a given splice-junction in multiple samples, which provides reproducible evidence that a given cryptic donor can be used by the spliceosome is intrinsic to ranking the top 4 events in 40K-RNA.
Randomization	For analysis of the cryptic-donor database, randomisation would be inappropriate as we wanted demographic information on database characteristics & features (i.e. prevalence of different types of mutations, algorithmic scores). Similarly for the analysis of naturally occurring human decoys- we wanted to analyse the prevalence, depletion, and proportion seen in 40K-RNA across the whole genome.
Blinding	Blinding was not relevant to our study as data collection consisted of compiling a database of variants, with specific ascertainment criteria, from public repositories. As such, no groups were allocated during data analysis but instead arose naturally from the data (e.g. AM-variants, CM-variants- according to the nature of the variant).

Reporting for specific materials, systems and methods

We require information from authors about some types of materials, experimental systems and methods used in many studies. Here, indicate whether each material, system or method listed is relevant to your study. If you are not sure if a list item applies to your research, read the appropriate section before selecting a response.

Materials & experimental systems

n/a	Involved in the study
☒	☐ Antibodies
☒	☐ Eukaryotic cell lines
☒	☐ Palaeontology and archaeology
☒	☐ Animals and other organisms
☒	☐ Human research participants
☒	☐ Clinical data
☒	☐ Dual use research of concern

Methods

n/a	Involved in the study
☒	☐ ChIP-seq
☒	☐ Flow cytometry
☒	☐ MRI-based neuroimaging
